# PTMselect: optimization of protein modifications discovery by mass spectrometry

**DOI:** 10.1038/s41598-019-40873-3

**Published:** 2019-03-12

**Authors:** Renaud T. Perchey, Laure Tonini, Marie Tosolini, Jean-Jacques Fournié, Frédéric Lopez, Arnaud Besson, Frédéric Pont

**Affiliations:** 1 LBCMCP, Centre de Biologie Intégrative, CNRS, Toulouse, France; 2 Centre de Recherches en Cancérologie de Toulouse, INSERM UMR1037, Toulouse, France; 3 Université Toulouse III: Paul-Sabatier, Toulouse, France; 4 ERL 5294 CNRS, Toulouse, France; 5 Université de Toulouse, Toulouse, France

## Abstract

Discovery of protein modification sites relies on protein digestion by proteases and mass spectrometry (MS) identification of the modified peptides. Depending on proteases used and target protein sequence, this method yields highly variable coverage of modification sites. We introduce PTMselect, a digestion-simulating software which tailors the optimal set of proteases for discovery of global or targeted modification from any single or multiple proteins.

## Introduction

Post-translational modifications (PTMs) play a crucial role in the regulation of all cellular processes in eukaryotic cells, and over 300 eukaryotic PTMs including phosphorylation, glycosylation, glycation, oxidation, are currently known^1^. PTMs are emerging as important markers of aging and age-related diseases such as Alzheimer’s and Parkinson’s diseases, cancer or diabetes^2^. Modified proteins are key regulators of signaling pathways involved in cellular homeostasis. Phosphorylations are the most frequent protein modification in cells and the human genome encodes 518 protein kinases, with an estimated 700,000 potential phosphorylation sites in eukaryotic cells^3^. Phosphate removal is catalyzed by protein phosphatases and 147 protein phosphatases have been described in human. Protein phosphorylation often triggers conformational changes that can affect all aspects of protein function, including enzymatic activity, protein stability, localization, interactions with other proteins and cellular components and are involved in the regulation of virtually all cellular processes^3^. An added complexity stems from frequent phosphorylation of proteins on multiple residues by different kinases. Identifying modified residues is therefore an important issue to understand this biochemical complexity.

A large number of PTMs in eukaryotic proteins have been identified by high-throughput mass spectrometry^1^ and recent advances in liquid chromatography mass spectrometry (LC-MS) have rendered PTM identification more amenable and sensitive.

To achieve the high sensitivity required for PTM analysis by LC-MS, proteins are cleaved into peptides. For its high specificity and ease of use, trypsin is predominantly used for protein digestion in MS^4^. However, trypsin misses out many PTM sites because all tryptic peptides do not have the optimal length^4,5^ for MS detection. This problem was overcome in large-scale protein analyses by parallel digestions with alternate proteases^4,5^, and corresponding protocols for six proteases were recently reported^6^. However, to achieve full coverage of PTMs in one protein, it requires six experiments, allowing up to 63 distinct combinations of [1–6] digestion panels. Also, the optimal protocol remains unknown until all of these combinations are performed and their respective products are compared. The problem becomes even more complex when optimizing experimental conditions for the analysis of multiple PTMs on a set of several proteins, in which case, the number of combinations is multiplied by the number of PTMs and the number of proteins. Moreover, limiting amounts of proteins, cost, time and efforts require keeping the number of experiments as low as possible. Several tools for PTMs prediction have been developed^7–11^ and were reviewed recently in^12–16^. However to our knowledge, these tools are not designed for - and do not allow - quickly discarding the large number of inappropriate protease combinations in PTMs studies by MS. New tools allowing the implementation of such MS proteomics developments are therefore acutely needed. With this aim, we designed PTMselect, a software dedicated to computationally identify the best protease combinations for optimal coverage of any global or targeted protein modifications from one or several proteins. Since phosphorylation is the most common PTM, and alterations of protein phosphforylations are major contributors to various pathologies such as Alzheimer’s, Parkinson’s and cancer^17^, we chose phosphorylation as an example to illustrate the capabilities of PTMselect in PTM discovery.

## Results and Discussion

PTMselect was optimized to eliminate the tedious work of manually sorting and selecting peptides to choose digestion settings before performing any MS-driven PTM analysis. This software is designed for (1) optimization of global coverage of protein PTM sites, (2) optimization of protein PTM sites coverage with the highest probability to be modified and (3) optimization of target PTM positions coverage.

**Figure 1:**
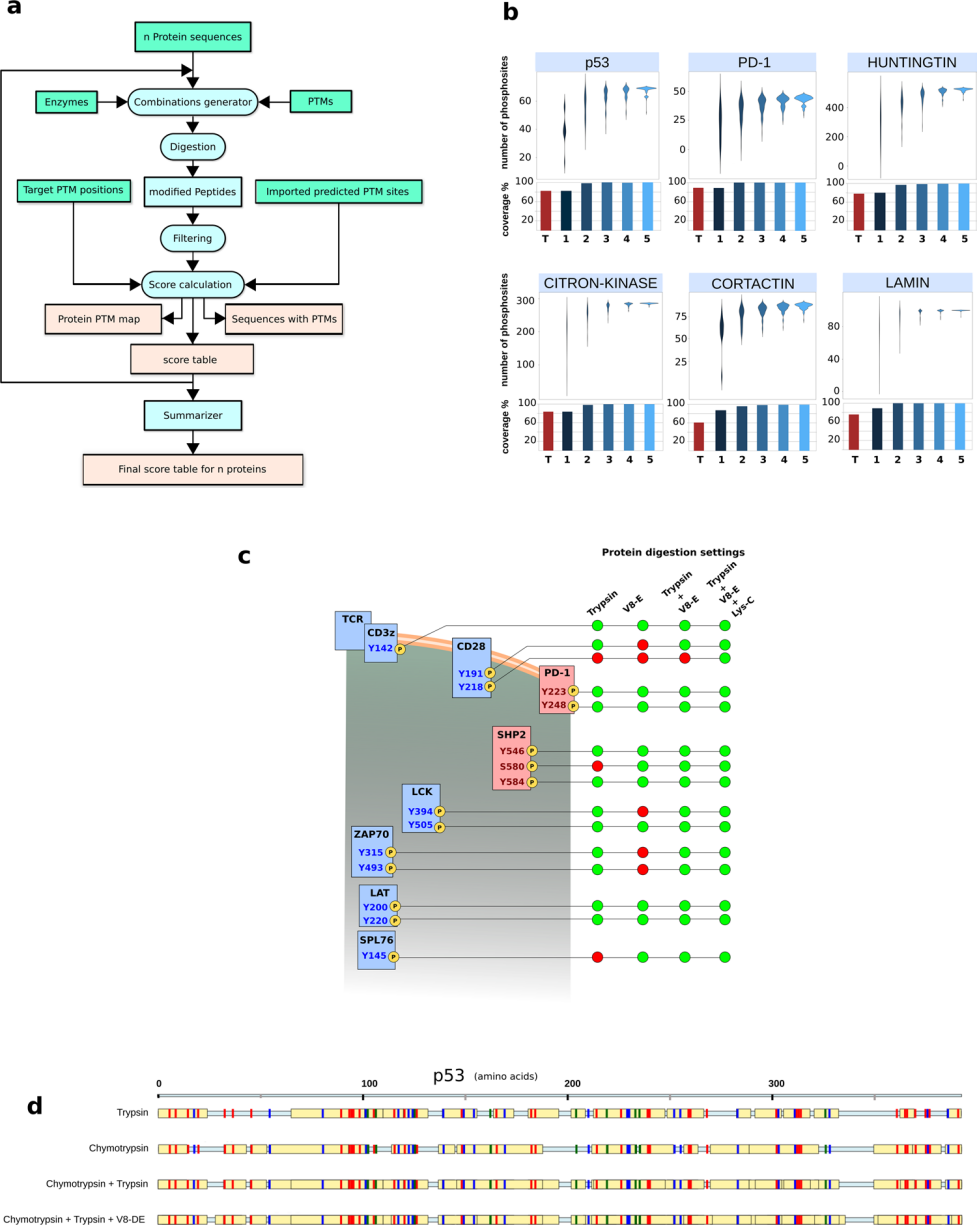
(**a**) Chart of PTMselect algorithm for PTMs discovery. Input files are in green boxes and output results in beige boxes. (**b**) PTMselect analysis of six protein sequences: p53, PD-1, Huntingtin, Citron-kinase, Cortactin and Lamin. For each protein, violin plots illustrate the range of phosphosites accessible by mass spectrometry, between worst and best digestion settings. The simulation was done with 8 enzymes and CNBr and [1–5] parallel digestions. Histograms show the percentage of coverage of all possible phosphosite positions with trypsin (red) and the best subsets of [1–5] proteases simulated by PTMselect (blue scale). (**c**) Example of simultaneous PTM analysis on multiple proteins with the targeted analysis of the TCR signaling pathway^17^. Stimulatory proteins are in blue and inhibitory proteins are in pink. Phosphosites of the proteins are yellow circles. The phosphosites of the TCR signaling pathway were used as targets in PTMselect and identifiable (green) and unidentifiable (red) phosphosites by a given set of digestion settings are illustrated for some of the best parallel digestions simulated by PTMselect: trypsin, V8-E and Lys-C. (**d**) Example of Phosphorylation map. The p53 protein was processed in PTMSelect and the maps obtained with trypsin and some of the best [1–3] protease combinations suggested by PTMselect are illustrated, showing a progressive increase of phosphosites coverage. Serine, threonine and tyrosine residues are in red, blue and green, respectively, and identifiable phosphopeptides are in yellow boxes.

PTMselect needs one or more protein sequences imported in Fasta format from protein databases, the identity of the amino-acids carrying PTMs, and the cleavages sites of the proteases (Fig. 1a). It computes all proteases combinations and the peptides released by these combinations. Peptides are further filtered by PTM sites and by size, compatible with LC-MS. A set of scores is then calculated, based on the number of PTM sites of the filtered peptides (Fig. 1a). All protein PTM site positions are considered equal and PTMselect returns the best digestion settings to maximize protein PTM coverage. Optionally, PTMselect can import a PTM position/score table from any prediction tool (Fig. 1a green box “imported predicted PTM sites”) and gives the best digestion settings to maximize protein PTM coverage of predicted positions with a dedicated score. Another option is to import a list of target PTM positions for one or more proteins. (Fig. 1a green box “target PTM sites”). With this information, PTMselect computes a target score and gives the best digestion settings to maximize PTM coverage of these specific protein sites. PTMselect also produces a graphical protein PTM map and a detailed result file featuring the sequence of all modified peptides and their position on the protein sequence. In case of multiple protein analysis, the software is able to merge the score table of each protein into a final score table.

PTMselect can analyze any number of proteins, PTM types and combinations. PTMselect simulations are based on protease specificities, peptide lengths and amino acids carrying the protein modifications. The eight proteases mostly used in MS (and CNBr) are available by default in PTMselect, but the user can add or remove proteases. PTMselect is compatible with any protein modifications, since only the amino acids linked to the modifications are necessary for the simulations, and can easily be changed in the PTMselect configuration file.

We chose protein phosphorylations to illustrate PTMselect capabilities. To estimate the real impact of PTMselect on the total number of phosphosites identifiable in a MS experiment, we selected six phosphoproteins with sizes ranging from 31.6 to 347.6 kDa (p53, PD-1, Huntingtin, Citron-kinase, Cortactin and Lamin) and we calculated the number of phosphosites released by the 8 default proteases in PTMselect (Arg-C, Asp-N, chymotrypsin, Lys-C, Lys-N, trypsin/P, V8-DE, V8-E) and CNBr on these six target proteins (Fig. 1b). Parallel digestion settings exhibit a wide range of efficiency to release phosphopeptides (Fig. 1b, violin plots). The histogram in Fig. 1b shows how detection of phosphosites can be improved by PTMselect. PTMselect improves phosphosite coverage in the six example proteins by up to 35% with 2 proteases and up to 37% with 3 proteases, when compared to use of trypsin alone.

As an example and to demonstrate the power of PTMselect in multiplex PTM analysis, we optimized phosophosite detection of the phosphoproteins involved in the TCR signaling pathway. This pathway is well described^17^ and involves 9 proteins with 15 known phosphosites illustrated in Fig. 1c. Using PTMselect, the best protease combination to simultaneously detect all regulatory sites was computed. With only 3 optimal digestion settings, all the phosphosites of the TCR signaling pathway were identified. The best digestion settings for MS analysis of the TCR signaling pathway were obtained in less than 30 sec of computing time.

The best compromise between the number of experiments and global (Fig. 1b) or targeted (Fig. 1c) phosphosite coverage is generally reached with 3 parallel digestions. As an exemple, Fig. 1d illustrates the progression of phosphosite coverage for p53 with 1, 2 and 3 parallel digestions.

PTMselect has not been initially designed for PTM cross-talk. But the software has the possibility to filter peptides matching a list of sequences or regular expressions. This feature can help PTM cross-talk studies. In Supplemental Fig. S4a, the unfiltered digestion pattern of Histone H3.1 is shown. In Supplemental Fig. S4b, a regular expression was used to select only peptides with the sequence “*K*_9_*STGGK*_14_” to study a potential cross-talk of acetylation between *K*_9_ and *K*_14_^18^. Since acetylation of Lysines can induce a missed cleavage, we show in Supplemental Fig. S5 that peptides filtration by regular expression “*KSTGGK*.” takes this missed cleavage into account.

**Figure 2:**
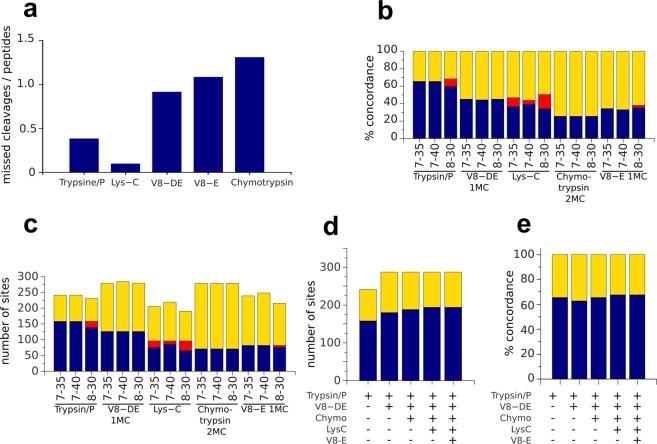
(**a**) Number of missed cleavages per peptide detected in LC-MS analysis of Citron-kinase digested with trypsin, Lys-C, V8-DE, V8-E and chymotrypsin. (**b**–**e**): Phosphosites of Citron-kinase detected by LC-MS after digestion with trypsin, V8-DE, Lys-C, chymotrypsin and V8-E compared with PMTselect simulations. Proteases producing a lot of missed cleavages (V8 and chymotrypsin) were simulated with one (V8) or two (chymotrypsin) missed cleavages (MC). Phosphosites exactly matching PTMselect simulation are in blue. Phosphosites predicted by PTMselect, but not observed (false positives) are in yellow. Phosphosites experimentally observed but not predicted by PTMselect (false negatives) are in red. (**b**) Effect of peptides sizes min and max [7–35], [8–30], [7–40] on the % of concordance between experimental and simulated phosphosites*. (**c**) Effect of peptides sizes min and max [7–35], [8–30], [7–40] on the number of phosphosites detected by LC-MS simulated by PTMselect*. (**d**) Effect of parallel digestion on the number of phosphosites detected by LC-MS simulated by PTMselect*. (**e**) Effect of parallel digestion on the % of concordance between experimental and simulated phosphosites*. *: same color code as in a).

To compare PTMselect simulations and experimental results, we analyzed by LC-MS, the effective phosphorylation sites of a very large protein (Citron-kinase, predicted MW 231.4 kDa) (Fig. 2). Five digestion settings were performed in parallel with chymotrypsin, Lys-C, trypsin, V8-DE and V8-E. In this experiment, the number of missed cleavages observed was highly variable. Chymotrypsin and V8 produced on average one missed cleavage per peptide (Fig. 2a). Lys-C produces less missed cleavages than trypsin, although the total number of peptides obtained with Lys-C was 51 and 125 with trypsin. So the best compromise between specificity and efficiency was obtained with trypsin.

To determine the impact of number of missed cleavages on the concordance between PTMselect simulations, and Citron-kinase phosphosites observed by LC-MS, a number of missed cleavage ranging from 0 to 3 were tested (Supplemental Fig. S3). Since Chymotrypsin and V8 produced many missed cleavages, the best results were obtained with 2 missed cleavages for chymotrypsin and 1 missed cleavage for V8 (Supplemental Fig. S3). Using these parameters, it was possible to reduce to zero the number of false negatives computed by PTMselect for chymotrypsin and V8 (e.g. phosphosites observed in LC-MS not predicted by PTMselect). No missed cleavages were required for trypsin and Lys-C. Indeed, 76% of tryptic peptide sequences with missed cleavages overlapped with peptides without missed cleavages, so the number of false negatives was always very low with trypsin. A large peptide, exceeding the default size filter setting of PTMselect, was obtained with Lys-C explaining a 4% false negative rate with all missed cleavage settings (Supplemental Fig. S3b).

Using the best missed cleavages settings, the effect of peptide filter sizes was explored for all phosphorylation sites of Citron-kinase. Simulations were done with 3 sets of peptides sizes [7–35], [7–40] and [8–30] (Fig. 2b,c). We found that trypsin was the most efficient protease with the best phosphosite concordance with the simulations (65%) (Fig. 2b) and the best phosphosite coverage (Fig. 2c). The best peptides size was [7–40] for all proteases (Fig. 2b,c).

Combinations of digestion settings were then simulated using the best filter size and missed cleavage parameters established for a single protease (Fig. 2d,e). To obtain good concordance and coverage of phosphosites, it was mandatory to include trypsin, the most efficient protease, in all digestion settings. The best compromise between the number of experiments and the number of Citron-kinase phosphosites observed experimentally was 3 digestions settings, as previously observed in theoretical simulations (Fig. 1b).

In all protease combinations including trypsin, the false negative rate obtained by PTMselect was null (Fig. 2d,e). Thus, PTMselect is very powerful to discard inappropriate digestion settings and this is especially useful for analysis of PTMs on specific sites. All simulated peptides may not be detected, as coverage of PTMs sites by MS is dependent on many factors. Such factors comprise sample preparation, protein amount, protein size, peptide MS sensitivity, peptide length, protease choice and digestion efficiency. Nevertheless, the concordance still ranged from 65.1 to 67.6% with all protease combinations using trypsin for Citron-kinase, a protein very difficult to analyze extensively with a total of 287 possible phosphorylation sites (Fig. 2e). This experiment also reveals to what extent PTMs discovery by LC-MS can benefit from parallel digestion settings. PTMselect, in combination with parallel protease digestions, should facilitate PTMs discovery or monitoring PTMs on specific sites of a protein set, such as a signaling pathway, by performing thousands of simulations within seconds. The very low false negative rate of results allows the user to quickly discard inappropriate protease combinations and spare sample time, efforts and costs.

## Methods

### PTMselect overview

PTMselect determines the optimal set of proteases to improve global coverage of protein modification discovery by MS analysis by simulating parallel digestions with all possible combinations of proteases. Four types of optimizations can be performed with PTMselect:

1. *Global* modified site coverage discovery for at least one protein: all modified sites are considered to have equal importance and PTMselect calculates the best digestion settings to obtain the largest number of modifications.
2. *Predicted* modified site coverage discovery for at least one protein: modified sites with the highest probability to be modified receive the highest scores. PTMselect computes the digestion setting to match the largest number of sites with a high probability to be modified.
3. *Targeted* modified site discovery for at least one protein: a list of target modification positions is given by the user for each protein. PTMselect optimizes the discovery of the largest number of modified sites in the lists or the total number of targeted proteins, i.e. the proteins with at least one target modification.
4. The last possibility is to combine *global*, *predicted* and *targeted* optimization for any number of proteins and any modifications.

PTMselect selects or rejects modified peptides of a digestion setting according to their lengths. Indeed, a mismatch between the *in silico* tryptic peptide distribution and optimal peptide length for successful mass spectrometry is always observed^5^. PTMselect performs simulations with a peptide length of 7 to 40 amino acids by default, which is a good initial setting for human cell analysis by MS in our and others experience^5^. This range can be adjusted by the user.

### PTMselect usability

PTMselect has been developed with usability and speed in mind.

The PTMselect basic tutorial (supplemental video PhosphoSelect_Basic_Tutorial_and_Install_v3.mp4) shows that PTMselect can be installed within minutes on MS Windows. The main task of the user is to download the protein Fasta files, then start PTMselect and enter the number of parallel digestions to simulate.

The PTMselect advanced tutorial (supplemental video VideoTutorial2_TCRpathway_v3.mp4) shows that the simulation of the best digestion settings for detection of the phosphorylations regulating an entire signaling pathway is easy too. All protein fasta files are copied in the fasta directory, and the target phosphosite files are simple text files with the positions of the targets sites in the protein sequence. Results are obtained within seconds.

Amino acids carrying the PTM can be easily changed. A unlimited number of amino acids can be targeted allowing the simultaneous optimization of detection of many modification sites with multiple modifications as well.

### PTMselect algorithm

#### PTMselect input

PTMselect processes proteins sequences in FASTA format (Fig. 1a). Two additional types of files can be optionally loaded and processed by PTMselect:

1. Prediction tables with modified positions and their prediction scores. These tables can be obtained from any prediction tool. PTMselect is compatible by default with PhosphoPICK^11^. For each phosphosite of a given peptide, PTMselect sums the phosphosite “combined-score” of PhosphoPICK to calculate the global predicted score of the peptide.
2. Lists of target modification site positions. These lists are text files containing known modification site positions mandatory for the biologist’s project, for example the phosphosites involved in a signaling pathway (Fig. 1c).

#### In silico protein digestion and peptide filtering

PTMselect asks the user to enter the maximum number n of parallel digestions he wants to simulate. PTMselect begins by calculating all combinations of n proteases starting from one ([1], [2],..[1, 2], [1, 3]..). Then, for each combination, it performs *in silico* parallel digestions of the protein. PTMselect uses by default 8 proteases and CNBr. This list can be reduced or increased if necessary. It then removes peptides without modification sites or outside the peptide length range.

#### Score calculation

PTMselect calculates five scores: maximal, electro transfer dissociation (ETD), collision induced dissociation (CID), predicted matched and predicted unmatched.

- The maximal score is the total number of modified sites in the protein.
- The ETD score is the total number of modified sites in the peptides after digestion and filtering. Indeed, any labile modified site can be attributed unambiguously by electro-transfer dissociation^19,20^.
- The CID score. Labile modified sites cannot always be attributed unambiguously when modified peptides are analyzed by collision induced dissociation^19,20^ because spectra are often dominated by large neutral loss peaks compromising reliable site-specific identification^21^. That is why PTMselect gives more weight to mono modified peptides in the CID score calculation.

The CID score of a peptide with n modified sites is:

$${p}_{CID}=\frac{1}{n}$$

The score of the entire protein with *k* modified peptides is:

$${P}_{CID}={\sum }_{i\mathrm{=1}}^{k}\,{p}_{CI{D}_{i}}$$

- The predicted matched score is the sum of each individual modified site score predicted by a prediction software for all peptides selected after digestion and filtering.
- The predicted unmatched score is the sum of each individual modified site score predicted by a prediction software for all peptides rejected after digestion and filtering.

#### Results output

The five modification scores (maximal, ETD, CID, predicted matched and unmatched) for each protease combination are exported in a table. PTMselect also calculates the number of mono-modified peptides, the number of target peptides accessible or not accessible and the corresponding lists of target site positions. A graphical map representing modified peptides and modification site positions is generated for each protease combination (Fig. 1d). The details of the modified sites in each peptide sequence and in the entire protein sequence are exported in a text file. PTMselect includes a summarizer able to process an unlimited number of score tables to calculate the sum of all the scores. When target modification sites are used, the summarizer builds a table with one target site by column. Thus, it is very easy to see which target sites are identifiable or not by a set of proteases.

#### PTMselect Benchmarks

Simulation time depends on the number of proteases and the size of the protein (cf. Supplemental Fig. S2). On a Linux 64 bit workstation with one CORE i7 processor the simulation time for 5 digestion settings out of 14, *i.e*. the simulation of 2379 protease combinations, was <6 sec for Lamin and <12 sec for Citron-kinase.

### Simulations of parallel proteases digestions

#### Protein sequences

We used six publicly available protein sequences to evaluate PTMselect (see Supplementary files). PD-1, p53, Huntingtin, Citron-kinase, Cortactin, and Lamin were chosen for their high phosphorylation level, size range, and biological relevance. Their fasta sequences were obtained from UniProt database^22^.

#### PTMselect simulations for six proteins

Parallel protease digestions were simulated for p53, PD-1, Huntingtin, Citron-kinase, Cortactin and Lamin using the default proteases list provided with PTMselect (8 proteases + CNBr). Up to five parallel digestions were simulated with a peptide size range from 7 to 40 amino acids (supplemental files).

#### PTMselect targeted analysis of the TCR signaling pathway

Fasta sequences of proteins of the TCR signaling pathway were downloaded from UniProt database^22^. Phosphosite positions for the proteins in this pathway where obtained from reference^17^ and PhosphoSitesPlus website^23^. For each protein, a text file containing the target site positions was created and used as input in PTMselect (Fig. 1a). Fasta files and target sites were processed together in PTMselect to produce a score table for each protein. In each score table, target phosphosites identifiable and unidentifiable by any digestion setting were listed. A combination of all score tables was computed automatically by PTMselect summarizer (Fig. 1a) in a summary table. The summary table was then sorted by number of target phosphosites identifiable in decreasing order, to identify the best digestion settings for MS analysis of the entire TCR pathway (supplemental files).

#### PTMselect prediction of multiple PTMs in a cross-talk example

Fasta sequence of protein H3.1 (*Mus musculus*) was obtained from from UniProt database^22^. The N-terminal methionine was removed from the sequence. Parallel protease digestions were simulated for H3.1 using the default proteases list provided with PTMselect (8 proteases + CNBr). To be able to analyse the cross-talk of K9 and K14 acetylation in the same peptide we set the number of missed cleavages to 3 for Lys-C, Lys-N and Trypsin. The number of missed cleavages for Chymotrypsin was 2, one for V8 and zero for Arg-C and Asp-N (Supplemental Fig. S5). To validate only peptides containing both K9 and K14 and not ending by K14 (we considered that Lysine acetylation induce a missed cleavage if Lysine is modified) a peptide filtration by regular expression was used. The regular expression “*KSTGGK*” was used to filter the peptides. The dot after KSTGGK implies that not only the KSTGGK sequence is present in the peptides but also that the peptides do not end by *K*_14_.

### Code availability

PTMselect was developed using the high performance cross-platform Julia language^24^ for numerical computing. Files can be accessed at (https://sites.google.com/site/fredsoftwares/products/ptm-select). A manual for using PTMselect to perform phosphosites basic and advanced search can be found in supplemental files. Peptides alignment tool, PepAlign, and lists comparison tool, nwCompare^25^, used to calculate the PTMs concordance are freely available at (https://sites.google.com/site/fredsoftwares/products/pepalign) and (https://sites.google.com/site/fredsoftwares/products/nwcompare-julia).

## Supplementary information

Supplemental information

supplemental video TCRpathway

Dataset 1

supplemental video PTMSelect_Basic_Tutorial_and_Install
