# Supplementary material for: PTMselect: optimization of protein modifications discovery by mass spectrometry
Source: Sci Rep. 2019 Mar 12;9:4181. doi: 10.1038/s41598-019-40873-3 (PMC6414543; doi:10.1038/s41598-019-40873-3)
Supplement: Supplementary file 1 — Supplemental information [file 41598_2019_40873_MOESM1_ESM.pdf]

Supplemental Information

PTMselect : optimization of protein modifications discovery by mass spectrometry

Authors:

Renaud Perchey, Laure Tonini, Marie Tosolini, Jean Jacques Fournié, Frédéric Lopez, Arnaud Besson & Frédéric Pont.

Supplemental Figures and Methods

1 Supplemental Methods

1.1 Protein production

HEK 293 cells were transfected by the calcium phosphate method for 48 h with a pcDNA6-Citron-Kinase-Myc-HisA construct encoding full-length CitK with C-terminal Myc and 6xHis tags. Cells were lysed in 600 μl of His wash buffer (50 mM Hepes pH 7.5, 250 mM NaCl, 0.05% Tween-20, 20 mM imidazole, 10% glycerol) complemented with 10 mM β-glycerophosphate, sodium fluoride and sodium orthovanadate, and with 10 μg/mL Aprotinin, Leupeptin and Pepstatin A. Cells extracts were sonicated for 10 s and centrifuged at 12500 g for 5 min. Lysates were filtered at 0.2 μm and loaded onto cobalt beads (Generon) in Generon Proteus 1 Step Batch columns (Gen-1SB08P) for 2 h at 4°C. Columns were spun 10 min at 750 g and washed twice with 20 mL of His wash buffer. CitK was eluted from beads by incubation with 500 μl elution buffer (50 mM Hepes pH 7.5, 150 mM NaCl, 0.05% Tween-20, 500 mM imidazole, 20% glycerol, 10 mM TCEP) for 15 min and centrifugation for 5 min at 750 g.

1.2 Protein preparation

Proteins were first reduced using Laemmli Buffer (40 mM DTT final) at 95°C for 5 min, then alkylated with iodoacetamide 90 mM for 30 min at RT in the dark. Next, protein migration was performed on 7.5% SDS PAGE and gels were stained by Coomassie Blue. A unique band was cut and washed several times in acetonitrile 100%, ammonium bicarbonate 100 mM and dried in vacuo. Gel pieces were rehydrated with $20ng/\mu l$ Trypsin prepared in ammonium bicarbonate 100 mM ; or with 400 ng Chymotrypsin prepared in 100 mM Tris HCl, 10 mM CaCl2, pH between 7-9 ; or with 400 ng Lys-C prepared in 25 mM Tris HCl, 1 mM EDTA, pH between 8-9 ; or with 400 ng Endoproteinase Glu-C (V8-E Protease) prepared in 25 mM ammonium acetate pH 4 ; or with 400 ng Endoproteinase Glu-C (V8-DE Protease) prepared in 25 mM ammonium carbonate pH 7.8, and submitted to in gel-digestion overnight at 37°C. Peptides were extracted and purified from gel and then subjected to mass spectrometry analysis.

1.3 Analysis of proteins by mass spectrometry

The peptides digested from citron kinase were measured on an SCIEX 5600+ TripleTOF mass spectrometer operated in DDA mode. A Dionex Ultimate 3000 nanoLC HPLC system and a Hypersil GOLD 150x0.32 mm column (Thermo Scientific), packed with C18 3 μm 175 Å material were used for peptide separation. For the HPLC method, buffer A was 0.1% (v/v) formic acid, and buffer B was 0.1% (v/v) formic acid, 90% (v/v) acetonitrile. The gradient was 4-45% of buffer B in 24 min with a flow rate of 5 $\mu L/min$. For MS method, a survey scan at the MS1 level (350-1600 m/z) was first carried out with 250 ms per scan. Then, the Top20 most intense precursors, whose charge states are 24 were fragmented. Signals exceeding 75 counts per second were selected for fragmentation and MS_2 spectra generation. MS_2 spectra were collected in the mass range 100-1600 m/z for 80 ms per scan. The dynamic exclusion time was set to 10 s.

1.4 Data analysis

To identify citron kinase peptides, profile-mode .wiff files from data acquisition were centroided and converted to mzML format using the AB Sciex Data Converter v.1.3 and submitted to Mascot[1] (version 2.5) database searches against UniProt[2] Swiss-Prot human database. ESI-Quad-TOF was chosen as the instrument, [trypsin/P, V8-E, V8-DE, Chymotrypsin, Lys-C] as the enzyme and up to 2 or 3 maximum missed cleavages were allowed. Peptide tolerances at MS and MS/MS level were set to be 20 ppm and 0.5 Da, respectively. Peptide variable modifications allowed during the search were phosphorylation of [STY], and oxidation of M, and peptide fixed modifications were carbamidomethylation of C. To calculate the false discovery rate (FDR), the search was performed using the decoy option in Mascot. Peptides with a $Pvalue \leq 0.01$ were selected. Peptides observed by MS were aligned on the Citron-kinase sequence using PepAlign (`https://sites.google.com/site/fredsoftwares/products/pepalign`). The PTMs position lists obtained with PepAlign for each digestion setting were then

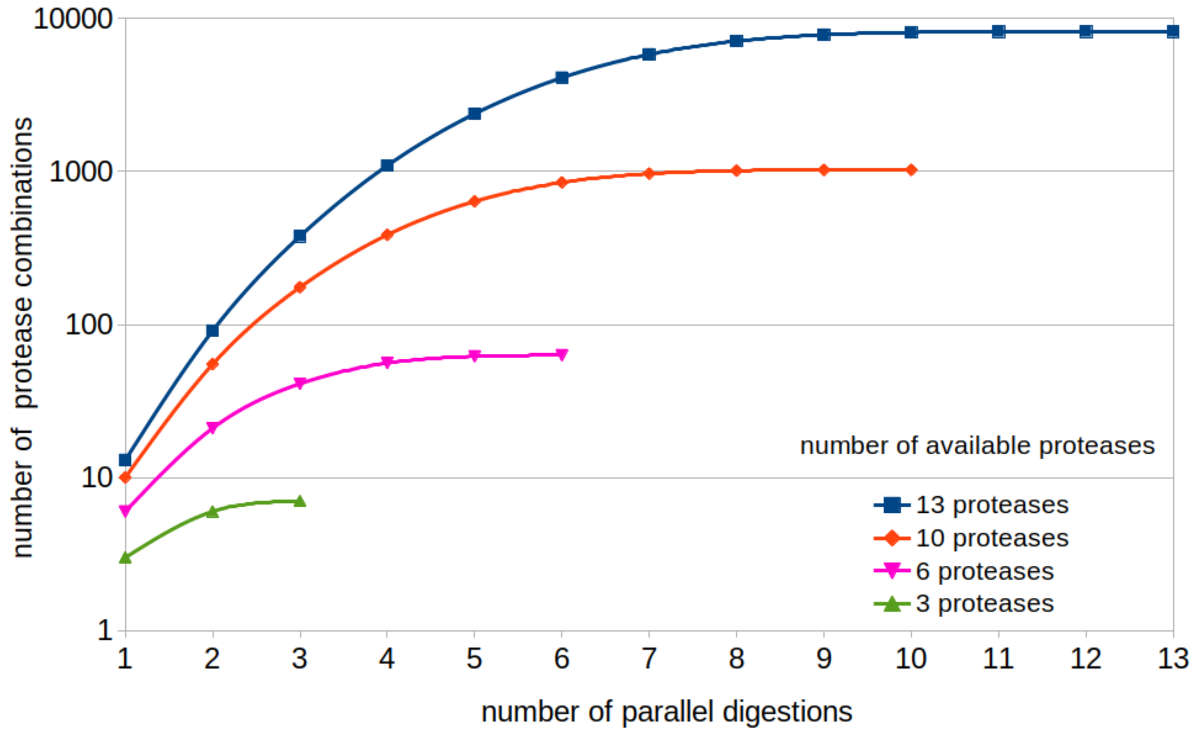

Supplemental Figure S 1: Cumulated number of possible protease (or digestion setting) combinations with 3, 6, 10 and 13 proteases versus the number of parallel digestions. For example with 3 proteases [a,b,c] (green line), the cumulated number of protease combination is 3 [a,b,c] with 1 digestion, 6 [a,b,c,ab,ac,bc] with 2 parallel digestions and 7 [a,b,c,ab,ac,bc,abc] with 3 parallel digestions.

compared with the simulated positions obtained by PTMselect using the comparison tool nwCompare[3] (`https://sites.google.com/site/fredsoftwares/products/nwcompare---julia`) to calculate the concordance.

References

[1] Perkins, D. N., Pappin, D. J., Creasy, D. M. & Cottrell, J. S. Probability-based protein identification by searching sequence databases using mass spectrometry data. *ELECTROPHORESIS: An International Journal* **20**, 3551–3567 (1999).

[2] Consortium, T. U. Uniprot: the universal protein knowledgebase. *Nucleic acids research* **45**, D158–D169 (2017).

[3] Pont, F. & Fournié, J. J. Sorting protein lists with nwcompare: A simple and fast algorithm for n-way comparison of proteomic data files. *Proteomics* **10**, 1091–1094 (2010).

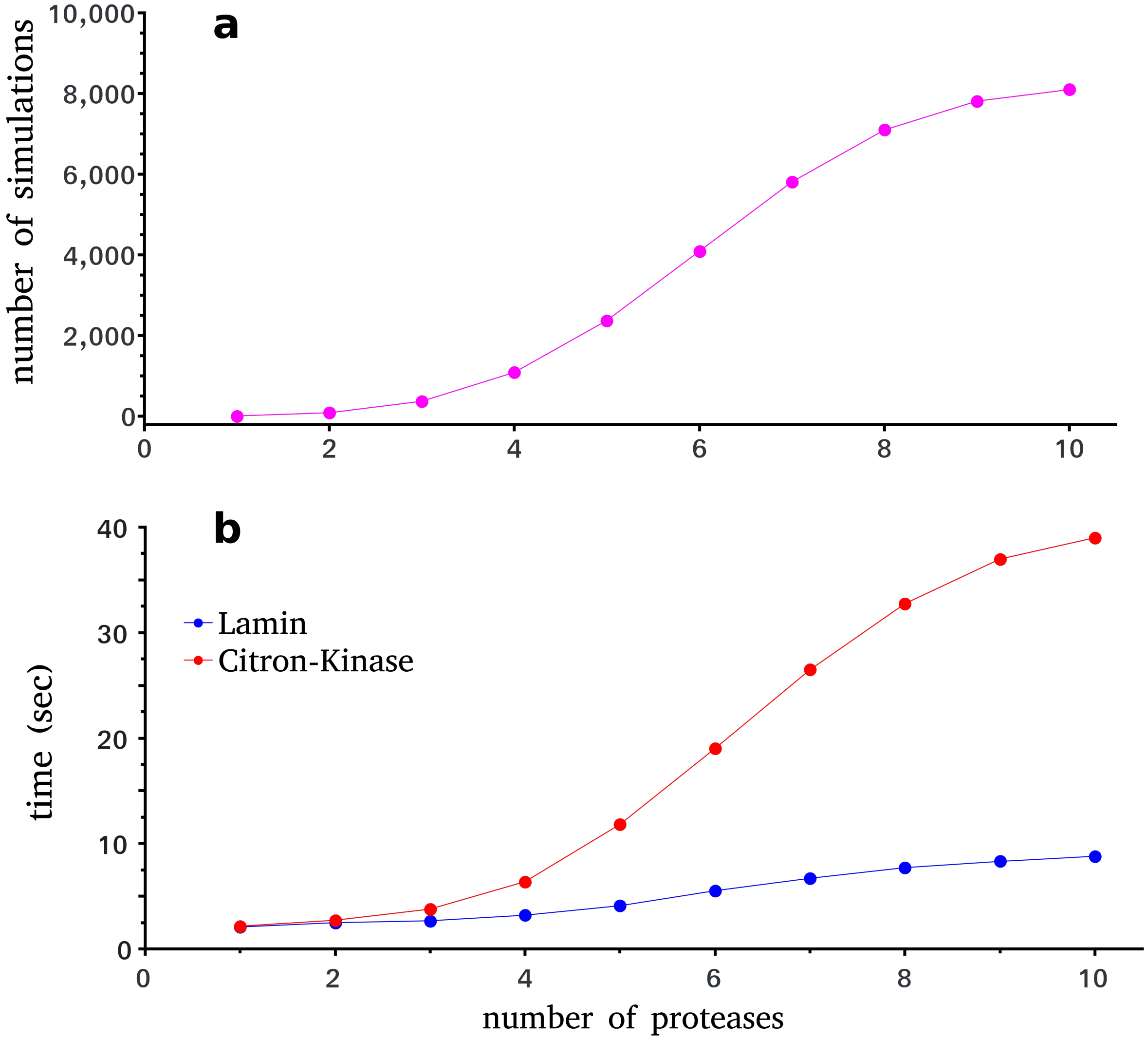

Supplemental Figure S 2: a) Number of simulations performed by PTMselect according to the maximal number of proteases in combinations. b) PTMselect performances on a Linux 64 bits workstation with a CORE™i7 processor. The simulation time from [1] to [1-10] proteases out of 13, was measured for Lamin and Citron-Kinase.

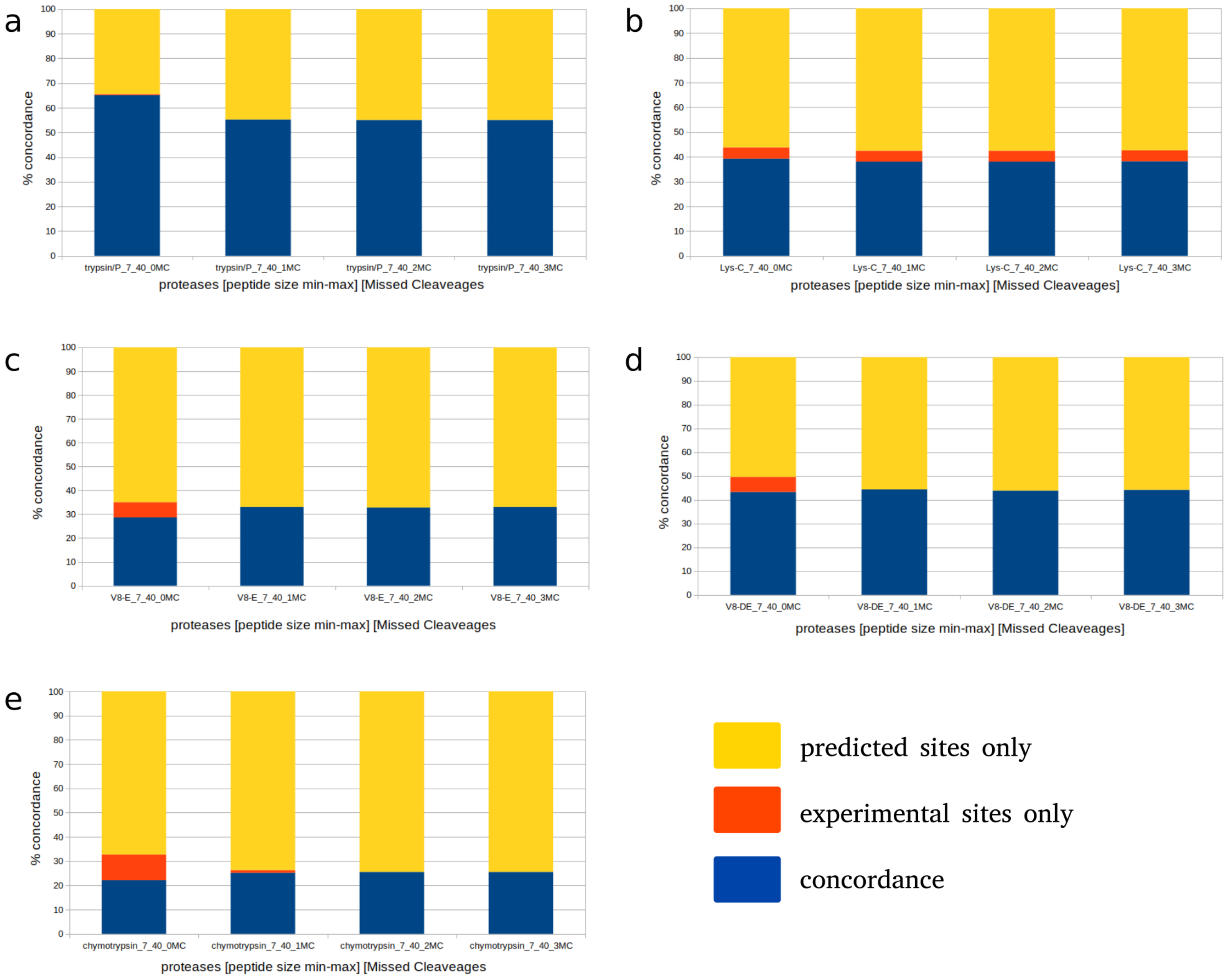

Supplemental Figure S 3: Phosphosites of citron kinase detected by LC-MS after digestion with trypsin (a), Lys-C (b),V8-E (c), V8-DE (d) and chymotrypsin (e) compared with PMTselect simulations. The graphs show the effect of the number of missed cleavages (MC) on the % of concordance between experimental and simulated phosphosites. The percentage of phosphosites exactly matching PTMselect simulation is in blue. The percentage of phosphosites predicted by PTMselect, but not observed (false positives) is in yellow. The percentage of phosphosites experimentally observed but not predicted by PTMselect (false negatives) is in red.

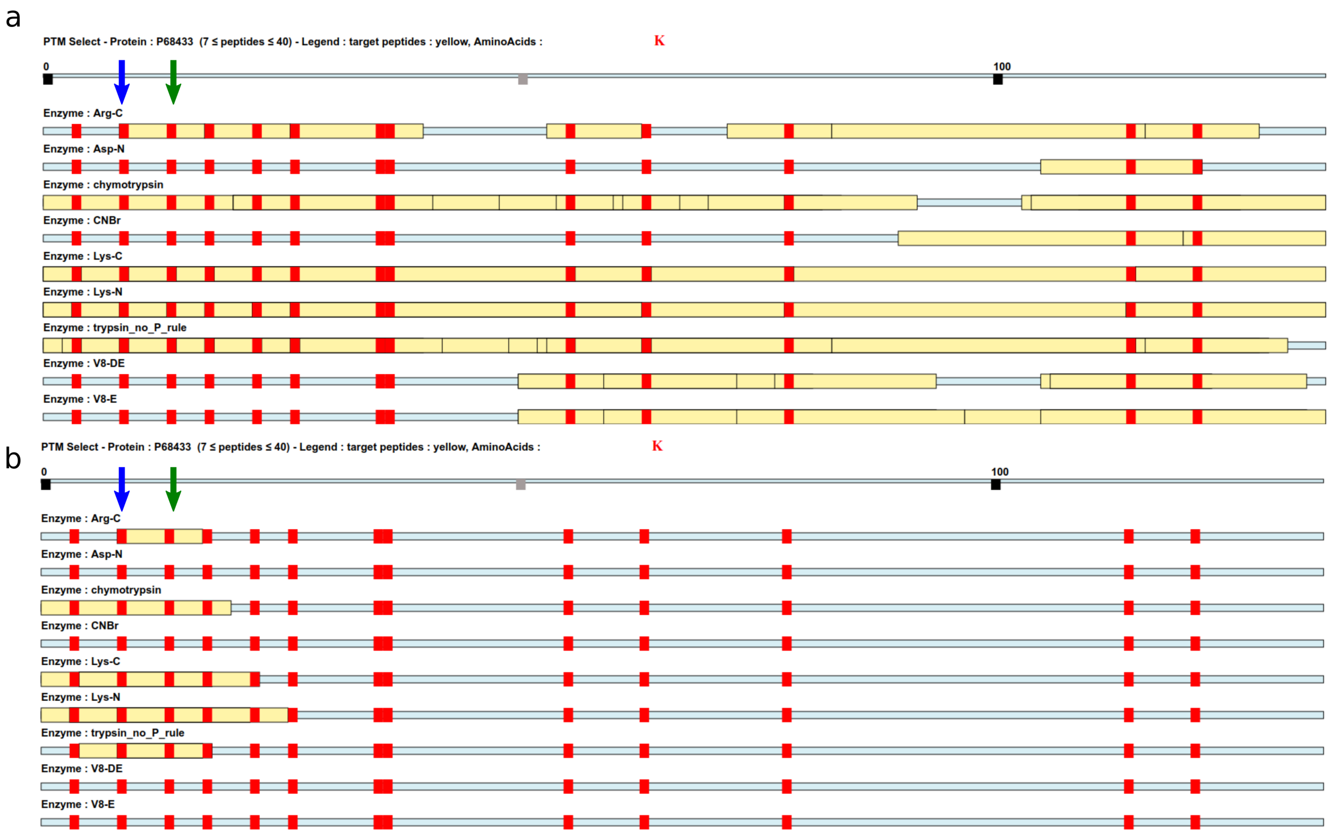

Supplemental Figure S 4: Prediction of multiple PTMs in a cross-talk example. Histone H3.1 (*Mus musculus*) digestion pattern obtained by PTMselect (yellow : target peptides) for prediction of the multiple PTMs possibly produced by a cross-talk between K9 (blue arrow) and K14 (green arrow). Red indicates K positions. a) Unfiltered digestion map, b) The digestion map filtered using the regular expression ”KSTGGK.” which selects peptides containing the contiguous sequence ”KSTGGK”. The dot at the end of this regular expression indicates that at least one amino acid must be present after the sequence ”KSTGGK”. Thus in this example, whenever K14 acetylation induces a systematic missed cleavage, peptides ending by ”K_9STGGK_{14}” are discarded. The list of peptides sequences produced by these digestions is provided in supplemental Figure S 5 . The N-terminal methionine was omitted from the sequence.

Enzyme	Target found	Missed Cleavages	Peptides filtered by regular expression
Lys-C	K9, K14	3	QTARKSTGGKAPRK, ARTKQTARKSTGGKAPRK, QTARKSTGGKAPRKQLATK
Lys-N	K9, K14	3	KSTGGKAPR, KQTARKSTGGKAPR, KSTGGKAPRKQLAT, ARTKQTARKSTGGKAPR, KQTARKSTGGKAPRKQLAT KSTGGKAPRKQLATKAAR
Chymotrypsin	K9, K14	2	ARTKQTARKSTGGKAPRKQL
Trypsin	K9, K14	3	KSTGGKAPR, QTARKSTGGKAPR, KSTGGKAPRK
Arg-C	K9, K14	0	KSTGGKAPR
CNBr		0	
V8-E		1	
Asp-N		0	
V8-DE		1	

Supplemental Figure S 5: Peptide sequences and parameters used for prediction of multiple PTMs by PTMselect in the cross-talk of K9 and K14 of Histone H3.1 (*Mus musculus*). The proteases used for digestion of protein H3.1 are shown in the column ”Enzyme”. K9 and K14 were designed as targets in PTMselect enabling quick selection of proteases releasing peptides with these sites (column ”Target found”). The maximum number of missed cleavages simulated for each protease is shown in column ”Missed Cleavages”. The regular expression ”$KSTGGK.$” was used to filter the peptides. The column ”Peptides filtered by regular expression” show the peptides validated by PTMselect after filtering. These peptides contains both K9 and K14 in their sequence. Moreover, it was assumed that a PTM acetylation at K14 induces a systematic missed cleavage, so peptides ending by K14 were removed by the regular expression ”$KSTGGK.$”. So, with the first five proteases of the table, it is possible to study both modifications of K9 and K14 and their potential cross-talk in the same peptides.
